# Recurrent Breast Abscesses in Type 2 Diabetes: A Diagnostic Challenge of Diabetic Mastopathy

**DOI:** 10.7759/cureus.90053

**Published:** 2025-08-14

**Authors:** Pulkit Mehrotra, Vengadakrishnan K, Nikitha Elizabeth Mathews

**Affiliations:** 1 Medicine, Sri Ramachandra Institute of Higher Education and Research, Chennai, IND; 2 General Medicine, Sri Ramachandra Institute of Higher Education and Research, Chennai, IND; 3 Internal Medicine, Sri Ramachandra Institute of Higher Education and Research, Chennai, IND

**Keywords:** breast abscess, diabetic mastopathy, lymphocytic mastitis, poor glycemic control, type 2 diabetes mellitus

## Abstract

Diabetic mastopathy (DMP) is a rare inflammatory disease of the breast that poses diagnostic challenges due to its clinical resemblance to breast carcinoma. Our patient, a 36-year-old woman with longstanding, uncontrolled diabetes, was encumbered with recurrent breast swellings that had become painful. Based on her clinical and laboratory findings, a provisional diagnosis of abscess was made, for which 'incision and drainage' was carried out. When repeated episodes yielded sterile cultures, a biopsy was made, thereby confirming a diagnosis of DMP. The patient was treated conservatively to optimize glycemic control and correct thyroid dysfunction. She was provided reassurance and was counselled regarding her condition to improve treatment adherence. Recurrence was not reported during subsequent follow-up visits.

Although its occurrence is rare, DMP should be considered a differential in the diagnosis of breast swellings in patients affected by diabetes mellitus. Timely identification of the disease prevents unnecessary surgical intervention and significantly reduces patient anxiety.

## Introduction

Diabetic mastopathy (DMP) is a rare condition that constitutes less than 1% of benign breast lesions [1]. It is classically associated with longstanding type 1 diabetes mellitus (T1DM) but is increasingly being reported in patients with type 2 diabetes mellitus (T2DM) [2]. First described in the 1980s, DMP typically presents as firm, fibrous, non-tender masses that mimic malignancy on clinical and radiological assessments [3]. Although pathogenesis remains unclear, chronic hyperglycemia, advanced glycation end-products, and autoimmune mechanisms are thought to be contributing factors [1].

Radiologically, DMP lacks pathognomonic features and may closely resemble breast carcinoma, often necessitating histopathological confirmation [4]. Management is conservative, with a focus on glycemic control. Surgical intervention is rarely indicated, except in cases of symptomatic pain or recurrent abscess formation. Important differential diagnoses to be considered are granulomatous mastitis, fibrotic tissue, and breast carcinoma [5]. The objective of this report is to highlight the diagnostic challenge of DMP in T2DM, a condition more typically associated with T1DM. This case is unique due to its presentation as recurrent sterile breast abscesses, its diagnostic overlap with malignancy, and its association with hypothyroidism despite negative autoimmune markers. By presenting this case, we aim to increase clinical awareness of DMP in T2DM and emphasize the role of histopathology in avoiding unnecessary surgical interventions.

## Case presentation

This case describes a 36-year-old female patient with a 12-year history of T2DM and hypothyroidism, who presented with a painful swelling in her left breast that began two days prior to her visit. She denied any associated systemic symptoms such as fever or weight loss. However, she reported having experienced a similar episode that occurred approximately six weeks prior, for which incision and drainage were carried out.

Her medication history included metformin 1000 mg twice daily and levothyroxine 50 µg daily, with no prior history of insulin use. Social history revealed that she was a non-smoker, did not consume alcohol, and had no history of recreational drug use. Gynecologic history indicated regular menstrual cycles, no history of pregnancy complications, and no family history of breast or ovarian malignancies.

A 4 × 4 cm tender, firm, poorly mobile, nodular subareolar mass was palpable in the lower outer quadrant of the left breast during physical examination. The overlying skin showed no significant changes such as erythema, dimpling, or peau d’orange, and axillary lymphadenopathy was absent. The contralateral breast was normal.

**Figure 1:**
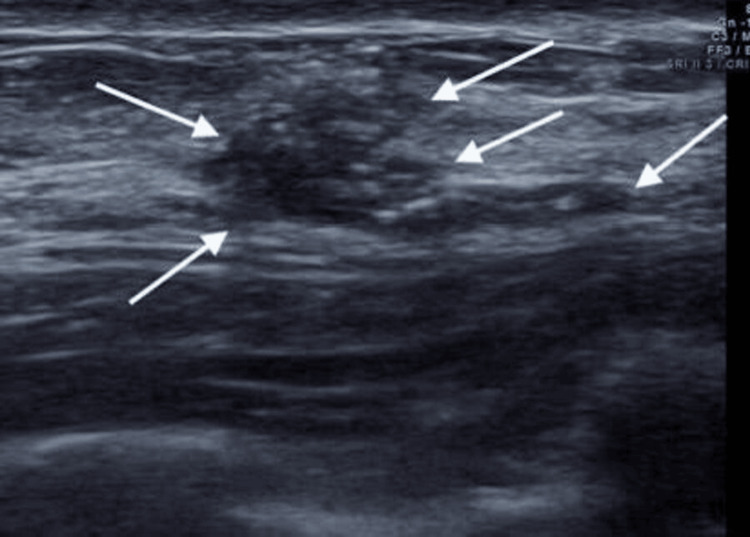
Histopathological section of breast tissue (H&E stain, 10× magnification) showing dense stromal fibrosis with prominent periductal and lobular lymphocytic infiltrates (white arrows), consistent with diabetic mastopathy.

Laboratory studies revealed leukocytosis, elevated inflammatory markers with normal hemoglobin levels, and an HbA1c of 9.0%, suggesting poor glycemic control (Table 1). Thyroid function tests showed elevated TSH (6.8 mIU/L) and low-normal free T4 (0.9 ng/dL), consistent with hypothyroidism. Ultrasound showed a Breast Imaging Reporting And Data System (BI-RADS) - 3 lesion, indicating that it was possibly benign (Figure 1).

**Table 1:** Laboratory findings indicating leukocytosis and poor glycemic control.

Parameter	Result	Normal Reference Range
Total Leukocyte Count (/mm³)	16,710	4,000–11,000
Hemoglobin (Female) (g/dL)	11.8	12.0–16.0
HbA1c (%)	9.0	< 5.7 (normal), 5.7-6.4 (prediabetic) >=6.5 (diabetic)

The findings suggested a probable abscess, for which the patient was administered insulin therapy to restore glycemic control during hospitalization (short-acting insulin on sliding scale), and she underwent incision and drainage under general anesthesia. Based on the provisional diagnosis of abscess, she was also started on empiric oral amoxicillin-clavulanate 625 mg twice daily for seven days and ibuprofen 400 mg three times daily for five days. The procedure yielded approximately 15 mL of purulent-appearing material, for which both Gram staining and culture results were negative.

**Figure 2:**
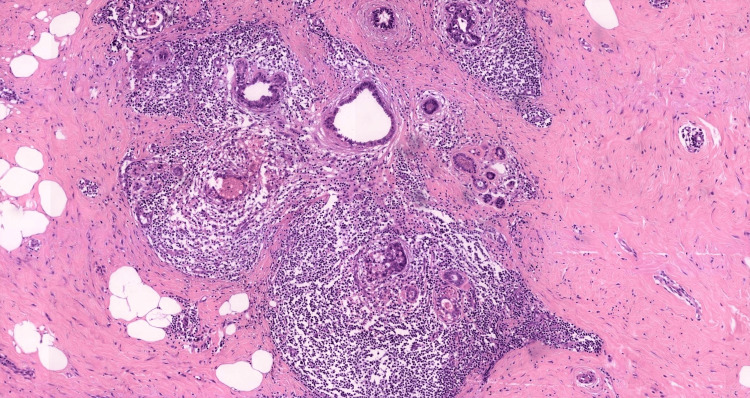
Histopathological section of breast tissue (H&E stain, 10x magnification) showing dense stromal fibrosis with prominent periductal and lobular lymphocytic infiltrates, consistent with diabetic mastopathy.

An incisional biopsy of the mass on the left breast was made. The sample measured approximately 2.5 × 1.8 × 1.2 cm and appeared as firm, grey-white fibrous soft tissue on gross examination. Histopathological analysis revealed dense stromal fibrosis with collagen deposition, with preservation of ductal and lobular structures and peripheral lymphocytic infiltrates, consistent with lymphocytic mastitis, confirming a diagnosis of DMP (Figure 2).

The firm, poorly mobile, nodular mass without skin changes or lymphadenopathy, combined with sterile cultures and characteristic histology, supported the diagnosis of DMP.

Insulin therapy was discontinued after discharge, and she was continued on oral hypoglycemic agents and thyroid supplements. The postoperative healing period was uneventful. She was followed up for six months, during which there was no recurrence of symptoms and glycemic control improved.

## Discussion

This case emphasizes the importance of considering DMP as a possibility in patients with breast masses and accompanying T2DM, particularly in conditions where glycemic control is suboptimal. Although uncommon in T2DM, DMP is being increasingly reported [6,7]. 

The patient's history of recurrent abscesses and accompanying sterile cultures suggested a non-infectious etiology. 

Radiologically, DMP remains a diagnostic dilemma, often mimicking malignancy. In our case, the BI-RADS 3 classification necessitated surgical intervention, although conservative management is generally preferred. 

Histopathological evaluation affirmed the diagnosis of DMP, demonstrated by lymphocytic infiltrates and dense stromal fibrosis consistent with typical features like lymphocytic ductitis and lobulitis with varying degrees of keloidal fibrosis, vasculitis, epithelioid fibroblasts, and lymphoid nodule formation [6]. 

Approach to diagnosis

The evaluation of suspected DMP begins with a detailed clinical history and examination, focusing on long-standing diabetes, autoimmune comorbidities, and recurrent or non-resolving breast lesions. Ultrasound is the first-line imaging tool but is non-specific. Mammography or MRI may be performed in selected cases but also lacks specificity. Given the overlap with breast carcinoma and infectious mastitis, core needle biopsy is essential for definitive diagnosis, demonstrating dense stromal fibrosis and periductal/perilobular lymphocytic infiltration.

A noteworthy observation in this case was the relevance of the history of hypothyroidism and its frequently associated autoimmune component despite a negative result obtained during our autoantibody screening [1,7]. A finding that supports the autoimmune model of pathogenesis put forth by Tomaszewski et al. [8]. It postulates that in sustained hyperglycemia, glycation of proteins leads to end products with antigenic properties, stimulating the production of autoantibodies and B-lymphocytes. A resultant release of cytokines contributes to the expansion of the epithelial stromal matrix and the differentiation of specialised epithelial cells that Tomaszewski et al. called epithelial fibroblasts. These epithelial fibroblasts embed themselves into the dense fibrous stroma [9,10], forming the typical histological features characteristic of DMP that were observed in our case. A similar autoimmune pathology is observed in other glandular disorders like Sjogren's syndrome, parotitis and Hashimoto's thyroiditis [11]. 

Another theory proposed by Seidman et al. [12] is based on isolated incidences of anti-insulin antibodies reacting with ductal epithelium. It suggests that exogenous insulin may promote the development of anti-insulin antibodies that trigger inflammation and lead to fibrosis and lymphocytic infiltration of the breast tissue [10]. However, this mechanism is unlikely to have occurred in our patient, given that she had no history of receiving exogenous insulin treatment to control hyperglycemia. 

The absence of insulin therapy in our case challenges Seidman's theory in our clinical context. The literature emphasizes the recurrent nature of DMP, which is attributed to ongoing glycemic dysregulation [13]. Active management is usually avoided due to its asymptomatic course and potential for recurrence, which may be ipsilateral, bilateral, or contralateral. The mainstay of treatment is medication for symptomatic relief, optimization of the patient's glycemic control, and addressing other metabolic imbalances, such as thyroid dysfunction, in our patient. Patient counselling plays an essential role in abating the fear of cancer that may arise in anyone who develops a lump in the breast [2]. Surgical intervention, though typically avoided, may be warranted, as in our case, when the patient is symptomatic. 

This case contributes to the expanding clinical spectrum of DMP in T2DM and highlights the importance of awareness among clinicians to provide appropriate treatment. 

## Conclusions

DMP, though rare, is an important differential in diabetic women presenting with breast masses. Its presentation in T2DM, especially accompanied by poor glycemic control and thyroid disorders, warrants careful consideration. Histopathology is essential for diagnosis, and conservative management, including strict glycemic control and patient education, remains the cornerstone of therapy. Timely identification can help prevent unnecessary surgical procedures and reduce the likelihood of recurrence. 
